# Once-daily, late-night, modified-release hydrocortisone rapidly normalizes menstrual cyclicity in women with non-classic congenital adrenal hyperplasia

**DOI:** 10.1093/hropen/hoag071

**Published:** 2026-08-05

**Authors:** Matthias K Auer, Lea Tschaidse, Orsela Dervishi, Pia Kruse, Martin Bidlingmaier, Sonja Kunz, Kathrin Bayer, Ulrike M Krämer, Hanna F Nowotny, Nicole Reisch

**Affiliations:** Medizinische Klinik and Poliklinik IV, Klinikum der Universität München, LMU München, Munich, Germany; Medizinische Klinik and Poliklinik IV, Klinikum der Universität München, LMU München, Munich, Germany; Medizinische Klinik and Poliklinik IV, Klinikum der Universität München, LMU München, Munich, Germany; Medizinische Klinik and Poliklinik IV, Klinikum der Universität München, LMU München, Munich, Germany; Medizinische Klinik and Poliklinik IV, Klinikum der Universität München, LMU München, Munich, Germany; Medizinische Klinik and Poliklinik IV, Klinikum der Universität München, LMU München, Munich, Germany; Medizinische Klinik and Poliklinik IV, Klinikum der Universität München, LMU München, Munich, Germany; Institut für Medizinische Psychologie, Center of Brain, Behavior and Metabolism (CBBM), Universität zu Lübeck, Lübeck, Germany; Medizinische Klinik and Poliklinik IV, Klinikum der Universität München, LMU München, Munich, Germany; Medizinische Klinik and Poliklinik IV, Klinikum der Universität München, LMU München, Munich, Germany

**Keywords:** 21-hydroxylase deficiency, late-onset, non-classic, fertility, fecundity, pregnancy, Efmody, hormonal control, metabolism

## Abstract

**STUDY QUESTION:**

Does late-night, modified-release hydrocortisone (MR-HC) improve biochemical control and restore menstrual regularity in women with non-classic congenital adrenal hyperplasia (NCCAH)?

**SUMMARY ANSWER:**

Once-daily MR-HC substantially improved biochemical androgen control and rapidly restored menstrual cyclicity in women with NCCAH, including those with longstanding irregular cycles.

**WHAT IS KNOWN ALREADY:**

While pregnancy rates in women with NCCAH are generally comparable to those of the general population, time to conception is often prolonged and assisted reproductive techniques are frequently required. Optimizing adrenal androgen suppression through the superior pharmacokinetic profile of MR-HC may shorten this interval and reduce the need for fertility interventions. So far, MR-HC has not been tested in NCCAH.

**STUDY DESIGN, SIZE, DURATION:**

Prospective observational cohort study conducted between September 2021 and November 2025 at a tertiary referral center and European Reference Network (Endo-ERN) hub for congenital adrenal hyperplasia. Thirty women with NCCAH were enrolled and followed for a median of 22.5 months (interquartile range [IQR] 8.25–36.75), with up to five study visits. Only women with at least 3 months of follow-up were included in the analysis.

**PARTICIPANTS/MATERIALS, SETTING, METHODS:**

The cohort comprised 30 women with NCCAH. At initiation of MR-HC, 20 women transitioned from conventional glucocorticoid therapy, and 10 were treatment-naïve. Sixteen women had an active desire to conceive, while 15 presented with irregular menstrual cycles or secondary amenorrhea at baseline.

**MAIN RESULTS AND THE ROLE OF CHANCE:**

Among 14 evaluable women, ovulatory function, defined as return of regular menstrual cycles or conception, was restored in 13 (92.6%) of women within 6 months of MR-HC initiation. One woman with concomitant hypothalamic hypogonadism remained amenorrhoeic and commenced hormone replacement therapy. Cycle normalization occurred at a median MR-HC dose of 10 mg (IQR 10–15 mg), with all but one woman receiving a single bedtime dose. Among 16 women seeking pregnancy, ten conceived within a median of 2.65 months (IQR 2.0–12.4) compared to 8 months (IRQ 1.0-24.0) in a historic cohort of NCCAH women before the approval of MR-HC (not significant). Among those seeking pregnancy, 10 women had been attempting to conceive unsuccessfully for a median of 28.5 months (IQR 12–46) before. Six women did not become pregnant during follow-up (13.5 months; IQR 6.25–23). The hydrocortisone equivalent dose (HCeq) at the time of conception was significantly lower under MR-HC (10.0 mg (IQR 10–12.5) vs. 16.9 mg (IQR 15–25), *P* = 0.02) in the historic cohort. Transition to MR-HC led to marked reductions in early-morning salivary 17-hydroxyprogesterone and serum testosterone, independent of prior glucocorticoid (GC) exposure. Metabolic effects were minimal: body weight decreased slightly, glycated hemoglobin (HbA1c) rose transiently, predominantly in treatment-naïve women, before returning to baseline. Blood pressure, fasting glucose, and lipid profiles remained stable.

**LIMITATIONS, REASONS FOR CAUTION:**

While the rapid normalization of, in part, long-standing menstrual irregularities was striking, the absence of a control group does not allow causal inferences, and the single-center design within a specialized tertiary setting may limit generalizability.

**WIDER IMPLICATIONS OF THE FINDINGS:**

A single nightly dose of MR-HC appears sufficient to achieve hormonal control and subsequent menstrual-cycle restoration in most women with NCCAH and may improve time to conception. These findings support MR-HC as a promising therapeutic option in the management of NCCAH.

**FUNDING:**

This work was supported by the Deutsche Forschungsgemeinschaft (Heisenberg Professorship 325768017, project 314061271 TRR205 to NR and FBCRC-1665 -515637292 to NR and UK). LT was supported by the LMU Munich funding scheme (FöFoLe) and by the IFCAH (International Fund Congenital Adrenal Hyperplasia) grant 2023. HN was supported by the Clinician Scientist Program RISE, supported by the Eva Luise und Horst Köhler Stiftung & Else Kröner-Fresenius-Stiftung (2019_KollegSE.03 to HN).

**DISCLOSURES:**

N.R. was Principal Investigator and consulted for Neurocrine Biosciences and Diurnal Ltd. L.T., M.K. A. und H.F.N. were Sub-Investigators for Neurocrine Biosciences and Diurnal Ltd.

**TRIAL REGISTRATION NUMBER:**

N/A

WHAT DOES THIS MEAN FOR PATIENTS? Women with non-classic congenital adrenal hyperplasia (NCCAH) produce certain hormones in their adrenal glands, especially during the night, that can disrupt the normal menstrual cycle and make it harder to become pregnant.

In this study, 30 women were treated with a new form of hydrocortisone that was taken once a day at bedtime. Unlike standard hydrocortisone, this treatment releases the medicine slowly during the night, so it more closely matches the body’s natural cortisol rhythm. This helps reduce the excess hormone production at the time when it is usually highest.

After starting treatment, almost all women with irregular periods developed regular menstrual cycles, and most women who wanted to become pregnant were able to do so. The treatment was well tolerated and did not appear to have major effects on weight, blood pressure, blood sugar, or cholesterol.

Although larger studies are still needed, these results suggest that this once-daily treatment may be a good option for women with NCCAH, especially for those with irregular periods or who are trying to conceive.

## Introduction

Congenital adrenal hyperplasia (CAH) due to 21-hydroxylase deficiency (21OHD) is the most common form of inherited adrenal disorder. It is characterized by impaired cortisol synthesis and a compensatory overproduction of adrenocorticotropic hormone (ACTH), resulting in adrenal androgen excess (Auer *et al.*, 2023).

The clinical spectrum of 21OHD is determined by the degree of residual enzymatic activity. Although the disorder exists along a continuum, it is conventionally divided into classical and non-classical forms (Auer *et al.*, 2023), classical CAH being a rare disease with cortisol deficiency and adrenal androgen excess already *in utero*, causing ambiguous genitalia in female newborns.

Non-classical CAH (NCCAH) is relatively common, having a prevalence of about 1:1000 in Caucasian populations (Speiser *et al.*, 2018) and representing the most important differential diagnosis of polyendocrine metabolic ovarian syndrome (PMOS, formerly known as PCOS) (New *et al.*, 2019). Individuals with NCCAH typically do not experience clinically relevant cortisol deficiency but females develop signs of hyperandrogenism at any time after birth, most typically in adolescents and young adults (Auer *et al.*, 2023). Symptoms may include hirsutism and menstrual irregularities or acne in females and premature pubarche and advanced bone maturation during childhood in both sexes.

While in classic CAH, glucocorticoid (GC) therapy is essential to replace deficient cortisol and to suppress excessive adrenal androgen production, women with NCCAH are often managed with oral contraceptives to achieve regular menstrual cycles and to control hyperandrogenism-related symptoms. However, in women who decline hormonal contraception or wish to conceive, GC therapy is frequently indicated (Carrière *et al.*, 2023) as even mild adrenal overproduction of androgens and progesterone can disrupt the hypothalamic–pituitary–gonadal (HPG) axis (Auer *et al.*, 2021), leading to irregular cycles or amenorrhea and prolonged time to conceive (New *et al.*, 2019; Hirschberg *et al.*, 2021; Carrière *et al.*, 2023; Auer *et al.*, 2024). Consequently, while pregnancy rates are comparable to those in the general population (Hirschberg *et al.*, 2021), time to conception in women with NCCAH is often prolonged and assisted reproductive techniques may be required (Guo *et al.*, 2022; Auer *et al.*, 2024). In an international multicenter study, we found that time to conception was more than 1 year in all phenotypes of CAH, including women with NCCAH (Auer *et al.*, 2024).

In NCCAH without clinically relevant lack of cortisol but still impaired negative feedback loop on the hypothalamic–pituitary–adrenal (HPA) axis, hormonal imbalance mainly occurs over night when the unopposed ACTH stimulation causes an increase of adrenal GC and androgen precursors while hormones often are balanced during the day. In this context, dexamethasone or prednisolone are often used as a therapeutic option due to their higher potency and long duration of action. Given at bedtime, both suppress ACTH secretion and thereby reduce adrenal androgen production through negative feedback on the hypothalamic–pituitary axis. Dexamethasone traditionally has been used mostly in NCCAH for this purpose. However, it also elevates glucocorticoid concentration in the early night, potentially causing metabolic disturbances, and in case of dexamethasone, it also traverses the placenta in case of pregnancy with potential fetal side effects.

The modified-release hydrocortisone (MR-HC) formulation (Efmody©) is the first oral GC designed to approximate the endogenous circadian cortisol profile more closely, thereby leading to improved hormonal balance, in particular in the early morning (Reisch and Auchus, 2024). Fertility was not systematically assessed in the phase III trial, but anecdotally a number of pregnancies among participants and partners with classic CAH were observed (Merke *et al.*, 2021), despite contraceptive recommendations. Patients with NCCAH were not included in the trial.

The efficacy of MR-HC in terms of improving hormonal control and fecundity in women with NCCAH therefore remains unexamined. To address this research gap, we report in the present study the first assessment of the use of MR-HC in this patient population. The primary endpoint was the effectiveness in terms of control of adrenal androgens and subsequent menstrual cycle normalization. The secondary endpoints included pregnancy and time to pregnancy (in those with a desire to conceive), cardiometabolic safety, and the long-term acceptance of this treatment.

## Materials and methods

### Subjects

**Figure 1.**
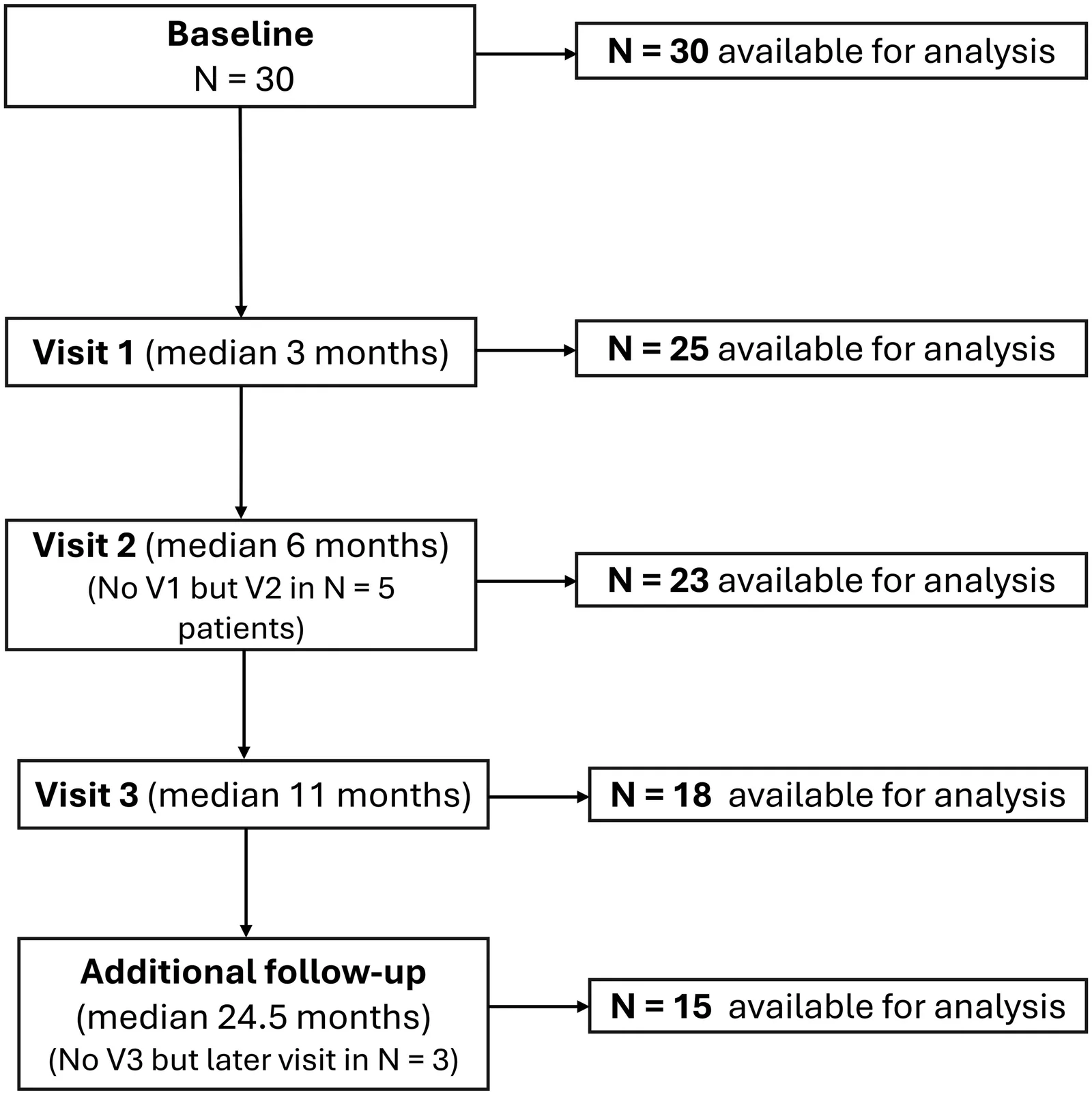
**Flow chart.** V1: visit 1; V2: visit 3, V3: visit 3.

Women with genetically and biochemically confirmed NCCAH due to 21OHD were recruited at the University Hospital of Munich through the Bio AI/DSD registry (ethical approval no. 19-558). All participants provided written informed consent. The current cohort was followed between September 2021 and November 2025, and a total of 30 women were enrolled. Of these, 20 patients were switched from conventional GC therapy to MR-HC, while 10 women were treatment-naïve at baseline. All participants had at least one follow-up visit ≥3 months after baseline. For comparison of time to pregnancy, a historic cohort of 20 women with NCCAH from the same center was included; data from this cohort had previously contributed to a multicenter study on pregnancy outcomes published elsewhere (Auer *et al.*, 2024). Patients were enrolled consecutively and prospectively followed after initiation of MR-HC. The median follow-up duration for the cohort was 22.5 months (interquartile range [IQR] 8.25–36.75). Follow-up visit 1 (V1), occurring after a median of 3 months (IQR 3–3.25), was available for 25 of 30 participants. Visit 2 (V2), conducted after 6 months (IQR 6–6.75), was available for 23 of 30 patients. Among these, 18 women completed both V1 and V2, seven had V1 only, and five had V2 only. Visit 3 (V3), performed after approximately 1 year (median 11 months, IQR 12–15), was available in 18 of 30 participants. Of these, 15 women had at least one additional later visit (median 24.5 months, IQR 24.5–37.5). In three further patients, V3 was not available, but a subsequent later follow-up visit was documented (Fig. 1).

Because recruitment was continuous and the present analysis was performed at a predefined data cut-off, follow-up duration varied between patients. Furthermore, follow-up visits were conducted as part of routine clinical care and therefore did not always occur exactly at the predefined study intervals.

Because pregnancy induces substantial physiological alterations, hormonal and metabolic assessments were evaluated only up to the time of first conception. Analyses of metabolic and hormonal changes were limited to the first three visits, as statistical power declined thereafter due to the smaller sample sizes. One woman without desire for future pregnancy discontinued MR-HC therapy and GC treatment in general after approximately 2 years as she no longer sought medical care. Two women had already conceived naturally prior to enrollment and had no further child wish.

### Laboratory measurements

Fasting blood samples were collected in the morning, 2–4 h after ingestion of the morning GC dose in women receiving morning GC therapy prior to switching. Serum total testosterone (T), sex hormone-binding globulin (SHBG), and 17-hydroxyprogesterone (17-OHP) were measured using chemiluminescent immunoassays on the iSYS platform (Immunodiagnostic Systems Ltd., Boldon, UK). Salivary 17-OHP concentrations were determined using a commercially available enzyme immunoassay (IBL International GmbH, Hamburg, Germany). Serum androstenedione (A4) was measured by chemiluminescent immunoassay on the LIAISON platform (DiaSorin S.p.A., Saluggia, Italy). Serum triglycerides and total cholesterol were measured using enzymatic colorimetric assays (Roche Diagnostics GmbH, Mannheim, Germany).

Salivary 17-OHP profiles were collected at five time points (before the morning dose, approximately 6:00–7:00 a.m.; 12:00 p.m.; 4:00 p.m.; 8:00 p.m.; and before the evening dose, approximately 10:00–11:00 p.m.) using standardized Salivette® collection devices (Sarstedt AG & Co. KG, Nümbrecht, Germany). Baseline salivary profiles were available for 23 women. Changes in salivary 17-OHP were analyzed up to visit 2 (approximately 6 months of follow-up).

Reference ranges were 0.4–3.4 ng/ml for A4, 14–69 ng/dl for T, 0.4–7.1% for the free testosterone index (fT), and 0.32–3.32 µg/l for serum 17-OHP. Given the high prevalence of irregular menstrual cycles in our cohort, the upper reference limit of the luteal phase and the lower reference limit of the follicular phase were used to encompass the physiological range observed in healthy women.

### Hydrocortisone equivalent dose

For the calculation of hydrocortisone-equivalent (HCeq) doses, the following conversion factors were applied: hydrocortisone = 1, prednisolone = 5, dexamethasone = 80 (Speiser *et al.*, 2018).

### Menstrual cycle definitions

Menstrual cycle disturbances were defined as cycle lengths shorter than 21 days or longer than 35 days. Secondary amenorrhea was diagnosed when menstrual bleeding had been absent for ≥3 months in previously regular cycles, or for ≥6 months in women with historically irregular cycles (Gordon *et al.*, 2017). No woman in this cohort was suffering from primary amenorrhea.

### Statistical analysis

All statistical analyses were performed using SPSS Statistics Version 23.0 (IBM Corp., Armonk, NY, USA). Graphical visualizations were generated with the help of ChatGPT Version 5.2 using Python 3.11 (McKinney, 2010) with the Matplotlib 3.7 (Hunter, 2007) and Seaborn 0.12 (Waskom, 2021) libraries. Descriptive data are presented as medians with IQRs and categorical variables as absolute and relative frequencies. Group comparisons for non-normally distributed variables were performed using the Mann–Whitney *U* test. Differences in categorical variables were analyzed using the chi-square test or Fisher’s exact test, as appropriate.

To evaluate longitudinal changes in outcome measures, including hormone concentrations and metabolic parameters, linear mixed-effects models were applied. Because hormone values (17-OHP, A4, T, fT) exhibited non-normal distributions, they were log-transformed prior to analysis. For each outcome, visit (time) and prior GC treatment were included as fixed effects together with their interaction. Repeated measures across visits were modeled at the patient level using an unstructured covariance matrix. For each model, estimated marginal means with 95% confidence intervals were obtained, and pairwise comparisons between visits and between prior treatment groups were performed using least significant difference tests. A *P*-value <0.05 was considered statistically significant.

## Results

Median age at baseline was 29.5 years (IQR 25.5–32.8). CYP21A2 genotypes can be found in the supplements (Supplementary Table S1). The most common non-classic allele was c.844G > T (p.Val282Leu), accounting for 35.0% of all alleles. Homozygous non-classical genotypes were observed in 20% of participants, while 53.3% were compound heterozygous for two non-classical pathogenic variants. The remaining 26.7% were compound heterozygous for a non-classical and a classic pathogenic variant.

### Medical history

Relevant comorbidities were documented in seven women: a history of anorexia nervosa with suspected hypothalamic hypogonadism (HH) (n = 1), a history of endometriosis and ectopic pregnancy with salpingectomy (n = 1), polycystic ovary morphology (PCOM, n = 1), hypothyroidism (n = 3), a woman with microprolactinoma receiving dopamine agonist therapy (n = 1) and who was normoprolactinemic at baseline and follow-up and one woman with type 1 diabetes mellitus (n = 1). The latter was excluded from fasting glucose analyses.

Among these women, only the woman with suspected HH and the woman with endometriosis and salpingectomy had a current child wish. The woman with HH was started on HRT after 1 year due to persisting hypogonadism despite adequate biochemical control, and natural conception was therefore not expected in the following.

Two women were taking metformin—one due to PCOM/insulin resistance, the other due to glucose intolerance; neither planned to conceive in the near future. One woman with insulin resistance started metformin during follow-up. This woman also wished to conceive. Three women desiring pregnancy were taking levothyroxine; thyroid-stimulating hormone (TSH) remained within the target range for women planning pregnancy (<2.5 μU/ml).

### Contraceptive history and reason for MR-HC treatment

At baseline, 5 patients (16.7%) were currently using hormonal contraception, and 12 patients (40.0%) had never used hormonal contraceptives in the past.

The primary indication for initiating MR-HC was a current or near-future child wish in 16 patients (53%), while the remaining 14 patients (47%) who were put on, switched to MR-HC due to cycle disturbances or hyperandrogenic symptoms or due to the idea that MR-HC might be beneficial in terms of glucocorticoid dose reductions (Merke *et al.*, 2021; Arlt *et al.*, 2025).

Among previous users, nine patients (30.0%) had discontinued hormonal contraceptives more than 12 months before initiating MR-HC, and two patients (6.7%) had stopped 3–12 months earlier. Only one woman (3.3%) had discontinued oral contraceptives within the 3 months preceding MR-HC initiation, such that discontinuation of oral contraception and initiation of MR-HC occurred in close temporal proximity. In one patient (3.3%), the contraceptive history was unclear (Table 1).

**Table 1.** Baseline characteristics of the study cohort.

	**Median (IQR)**
Median follow-up (months)	22.5 (8.25–36.75)
Visit 1 (months)	3 (3–3.25)
Visit 2 (months)	6 (6–6.75)
Visit 3 (months)	11 (12–15)
Visit 4 (months)	24.5 (24.5–37.5)
Age (years)	29.5 (25.5–32.8)
Weight (kg)	66.5 (59.2–77.3)
BMI (kg/m²)	25.1 (20.9–29.8)
**Previous glucocorticoid therapy**	**N (%)**
None	10 (33.3%)
Hydrocortisone	8 (26.7%)
Morning only	3 (37.5%)
Morning and noon	2 (25%)
Three times	3 (37.5 %)
Prednisolone	8 (26.7%)
Morning only	2 (25 %)
Evening only	0 (%)
Morning and evening	6 (75 %)
Dexamethasone (evening only)	4 (13.3%)
**Pregnancy-related parameters**	**N (%)**
Pregnancy during follow-up	10 (62.5%)
Time trying to get pregnant prior to MR-HC (months)*	28.5 (12–46)
Time to pregnancy on MR-HC (months)*	5.75 (2.1–12.4)
Prior ART	2 (12.5%)
**Glucocorticoid dosing**	**Median (IQR)**
HCeq at baseline (mg)	15.0 (12.2–17.8)
HCeq at baseline (mg/m²)	8.1 (5.8–9.82)
Initial Efmody dosage (mg)	10 (10–15)
HCeq initial Efmody dosage (mg/m²)	6.01 (5.57–9.95)
Dose difference after switch (mg)	−5.0 (−6.3 to −3.8)
**Switching pattern**	**N (%)**
Lower dose	9 (30.0%)
1:1 dose	9 (30.0%)
Higher dose	2 (6.7%)
No prior therapy	10 (33.3%)
**Relevant comorbidities**	**N**
History of anorexia nervosa/suspected hypothalamic hypogonadism	1
PCOM	1
Hypothyroidism (substituted)	3
Microprolactinoma (normoprolactinemic)	1
Diabetes mellitus type 1	1
Endometriosis + History of ectopic pregnancy with salpingectomy	1
**Menstrual cycle at baseline**	**N (%)**
Regular cycle	10 (33.3%)
Irregular cycle	11 (36.6%)
Amenorrhea	4 (13.3%)
Hormonal contraception	5 (16.7%)
**History of hormonal contraception**	**N (%)**
At baseline	5 (16.7%)
Never used	12 (40.0%)
>12 months before MR-HC	9 (30.0%)
3–12 months before MR-HC	2 (6.7%)
<3 months before MR-HC	1 (3.3%)
History unclear	1 (3.3%)

* Ten women had been attempting to conceive before switching to MR-HC, in two women, the switch to MR-HC coincided with the onset of an active desire to conceive, and in four women attempts to conceive were initiated after the switch.

IQR, interquartile range; HCeq, hydrocortisone-equivalent dose; MR-HC, modified-release hydrocortisone; PCOM, polycystic ovarian morphology.

### Prior GC treatment and initial MR-HC dosing

One third of the cohort (n = 10, 33.3%) had not received any GC therapy prior to MR-HC. Among previously treated patients, immediate-release hydrocortisone had been used by eight women (26.7%), another eight (26.7%) were treated with prednisolone, and four patients (13.3%) were on dexamethasone (Table 1).

At baseline, the HCeq dose was 15.0 mg (IQR 12.2–17.8), corresponding to 8.1 mg/m² (IQR 5.8–9.82) (Table 1). Patients were switched to MR-HC with a median dose of 10 mg (IQR 10–15), equivalent to 6.01 mg/m² (IQR 5.57–9.95).

The starting dose of MR-HC was determined by the treating physician and was primarily based on the patient’s estimated HCeq dose. However, an exact 1:1 conversion was not always feasible because the calculated hydrocortisone-equivalent dose of the previous regimen did not always correspond to the available modified-release hydrocortisone tablet strengths (5 mg and 10 mg), requiring rounding to the nearest achievable dose.

Compared to the previous HCeq dosing, nine patients (30.0%) were transitioned to a lower dose (≤2.5 mg), nine (30.0%) underwent a 1:1 dose switch, and two women (6.7%) were initiated on a higher dose (≤2.5 mg). Three patients with relatively high baseline HCeq doses (17.5–22.5 mg) were intentionally switched to a lower dose (10–15 mg). All women except two received a once-daily evening dose; two women took an additional 5 mg in the morning.

### Biochemical control at baseline

As expected, women without prior GC therapy showed significantly higher rates of elevated adrenal androgens and androgen precursors at baseline. Compared with previously treated patients, untreated participants had a higher proportion of serum 17-OHP above the female reference range (60% vs. 15%, *P* = 0.035), A4 (70% vs. 15%, *P* = 0.012) and T (100% vs. 26.3%, *P* < 0.001) (Supplementary Fig. S1).

### Dose adaptation during follow-up

Compared with the initial MR-HC dose prescribed immediately after switching from conventional glucocorticoids, the MR-HC dose at the last available follow-up visit was increased by 5 mg in four women, while it was decreased by 5 mg in another four women. In the remaining women, the total daily dose remained stable. Thus, in total, changes between baseline and the last available visit were non-significant (*P* = 0.999).

### Biochemical control

**Figure 2.**
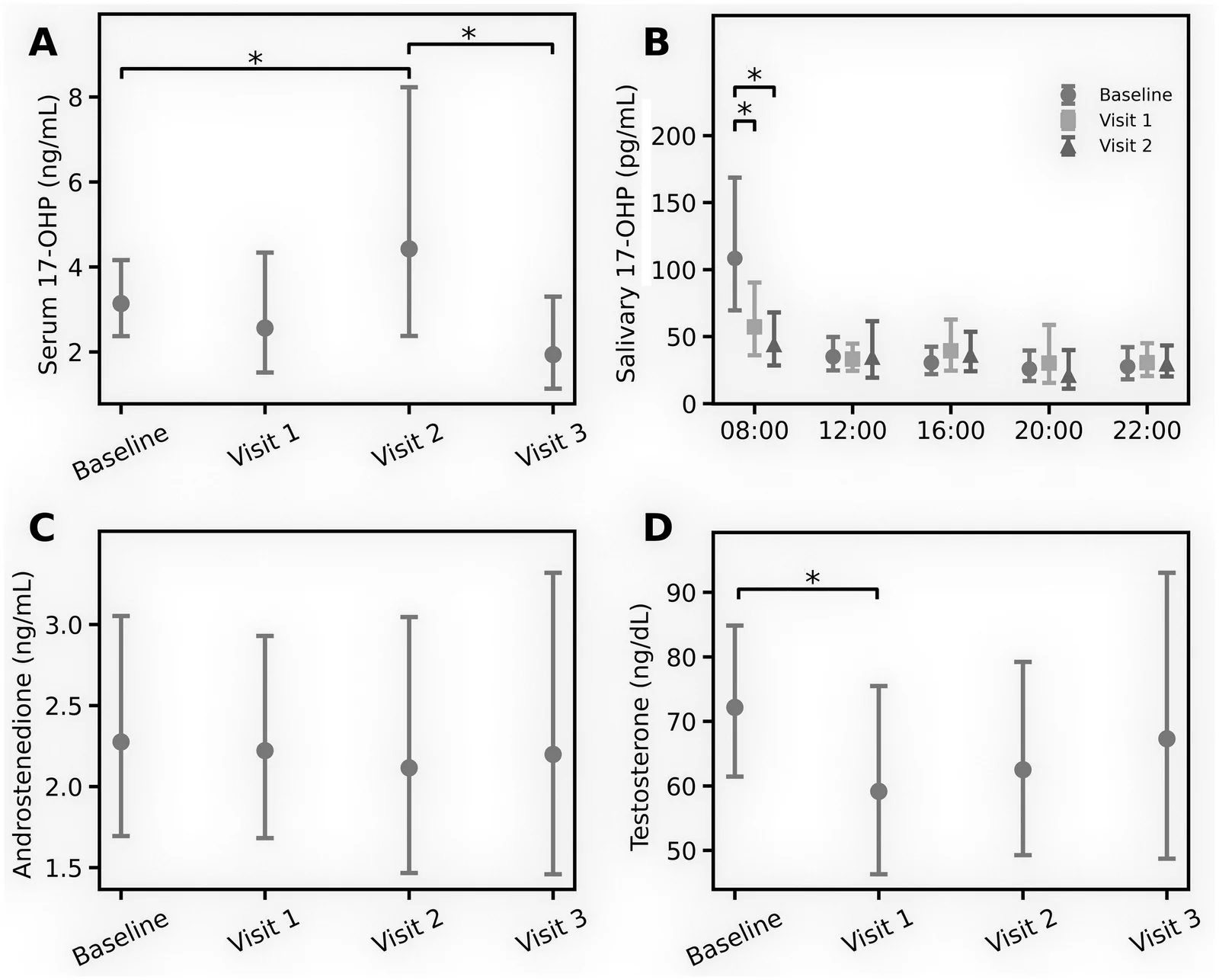
**Hormonal changes over time after initiation of modified-release hydrocortisone**. (**A**) Serum 17-hydroxyprogesterone (17-OHP), (**B**) salivary 17-OHP profiles, (**C**) serum androstenedione, and (**D**) serum testosterone. Mixed-effects models included visit (time), prior glucocorticoid treatment, and their interaction as fixed effects. Repeated measures were accounted for at the patient level using an unstructured covariance matrix. Points represent estimated marginal means and error bars indicate 95% confidence intervals; asterisks denote significant pairwise comparisons (P < 0.05). In panel B, the x-axis represents the predefined time points of salivary sample collection (08:00, 12:00, 16:00, 20:00, and 22:00). Circles represent baseline measurements, squares represent visit 1, and triangles represent visit 2. 17-OHP, 17-hydroxyprogesterone.

Mixed-model analysis of serum 17-OHP showed a significant effect of time (*F*(3,18.0) = 4.06, *P* = 0.023) as well as a significant time × pretreatment interaction (*F*(3,18.0) = 4.55, *P* = 0.015) Pairwise comparisons demonstrated that concentrations at visit 4 were significantly lower than at baseline (*P* = 0.032) and visit 3 (*P* = 0.011) (Fig. 2A).

Salivary 17-OHP in the early morning showed a significant effect of time (*F*(2,18.0) = 4.86, *P* = 0.021), whereas neither prior GC treatment (*F*(1,19.5) = 0.18, *P* = 0.672) nor the time × pretreatment interaction (*F*(2,18.0) = 0.18, *P* = 0.838) was significant. Pairwise comparisons demonstrated significantly lower early morning salivary 17-OHP concentrations at visit 1 (*P* = 0.026) and visit 2 (*P* = 0.014) compared with baseline (Fig. 2B).

For A4, there was no overall effect of time (*F*(3,13.6) = 0.07, *P* = 0.973). However, a significant interaction between time and prior GC treatment was observed (*F*(3,13.6) = 3.92, *P* = 0.033), indicating that changes over time differed between previously untreated and pretreated women. The overall difference between treatment groups showed a statistical trend but did not reach significance (*F*(1,26.4) = 4.10, *P* = 0.053) (Fig. 2C).

Serum testosterone revealed a significant effect of time (*F*(3,16.1) = 3.96, *P* = 0.027), a significant overall effect of prior GC treatment (*F*(1,26.5) = 5.99, *P* = 0.021), and a significant time × pretreatment interaction (*F*(3,16.1) = 7.23, *P* = 0.003). Pairwise comparisons indicated a significant decline from baseline to visit 1 (*P* = 0.006), whereas differences between the other visits were not significant (Fig. 2D).

### Menstrual cycle at baseline and during follow-up

**Figure 3.**
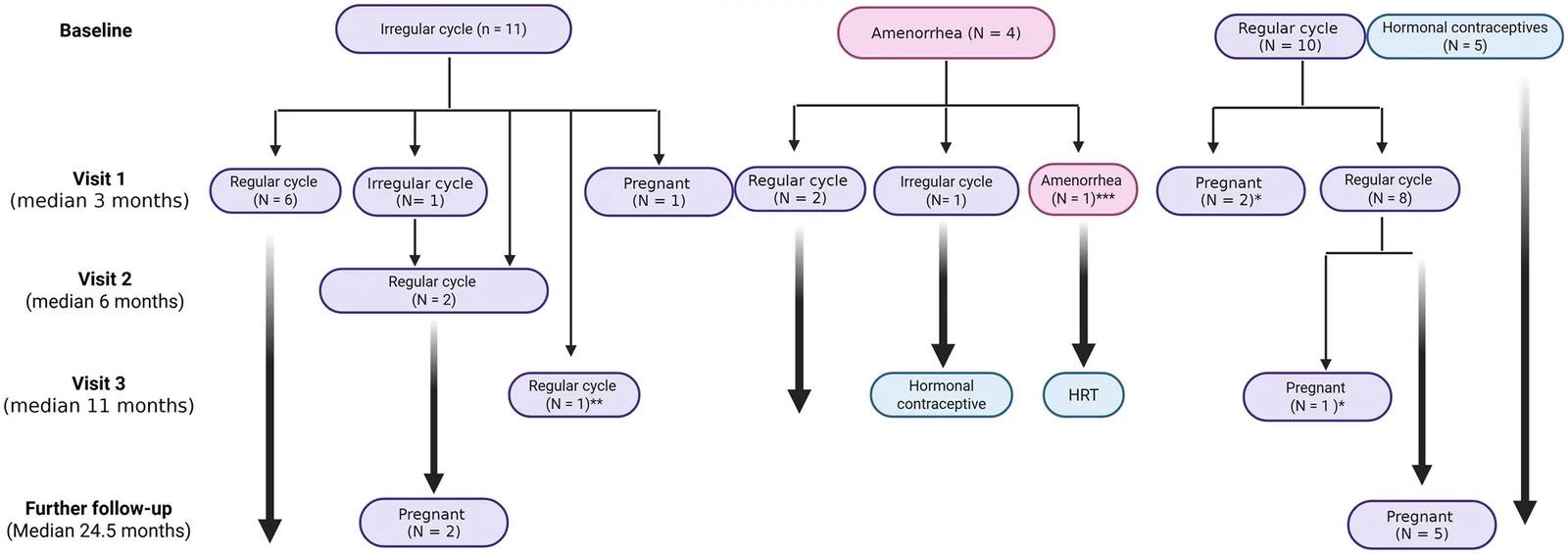
**Changes in menstrual cycle patterns and pregnancies**. The chart illustrates the clinical course of 30 women with non-classic congenital adrenal hyperplasia across four predefined visits (median follow-up 24.5 months). Three groups of patients are shown according to their baseline menstrual status: left, women with an irregular menstrual cycle (n = 11); middle, women with secondary amenorrhoea (n = 4); and right, women with a regular menstrual cycle (n = 10) or using hormonal contraceptives (n = 5). Purple boxes indicate regular cycle, irregular cycle, or pregnancy; pink boxes indicate secondary amenorrhoea; and blue boxes indicate hormonal contraception or hormone replacement therapy (HRT). Created in BioRender. Auer, M. (2026) https://BioRender.com/zmob85l. *One woman with a miscarriage in early pregnancy got pregnant again. **Information on menstrual cycle was missing in between. ***Hypothalamic amenorrhea due to a history of anorexia nervosa.

At baseline, regular menstrual cycles were reported by 10 women (33.3%), and 11 (36.6%) had irregular cycles. Amenorrhea was present in four women (13.3%). Five women (16.7%) were using hormonal contraceptives throughout the observation period (Fig. 3).

Of the 11 patients with an irregular cycle at baseline, 6 had regained a regular cycle until visit 1 after a median of 3 months, one became pregnant within the first 3 months, another two had regained a regular cycle at visit 2 (one of these had no visit at 3 months) and the last women by visit 3 (information on menstrual cycle was missing in between).

Of the four women with secondary amenorrhea, two had regained a regular cycle at visit 1, one regained an irregular cycle by visit 1 and was put on a hormonal contraceptive pill during follow-up. The last patient with hypothalamic amenorrhea remained amenorrheic and received HRT during follow-up at the third visit after 12 months.

Overall, when considering return of regular menstrual activity—including pregnancy as a marker of restored ovulatory function—13 of 14 women (92.6%) experienced a treatment response within a maximum of 6 months when excluding the one woman with hypothalamic amenorrhea who was not expected to regain a menstrual cycle due to optimization of adrenal androgen control. Cycle normalization was achieved with a median of 10 mg MR-HC (IQR 10–15). Except for one patient, all of these women received a single evening dose.

Improvement in menstrual cycle regulation was equally efficient in treatment-naïve patients and in those with prior GC therapy (Supplementary Fig. S2).

#### Hormonal control and menstrual cycle

**Figure 4.**
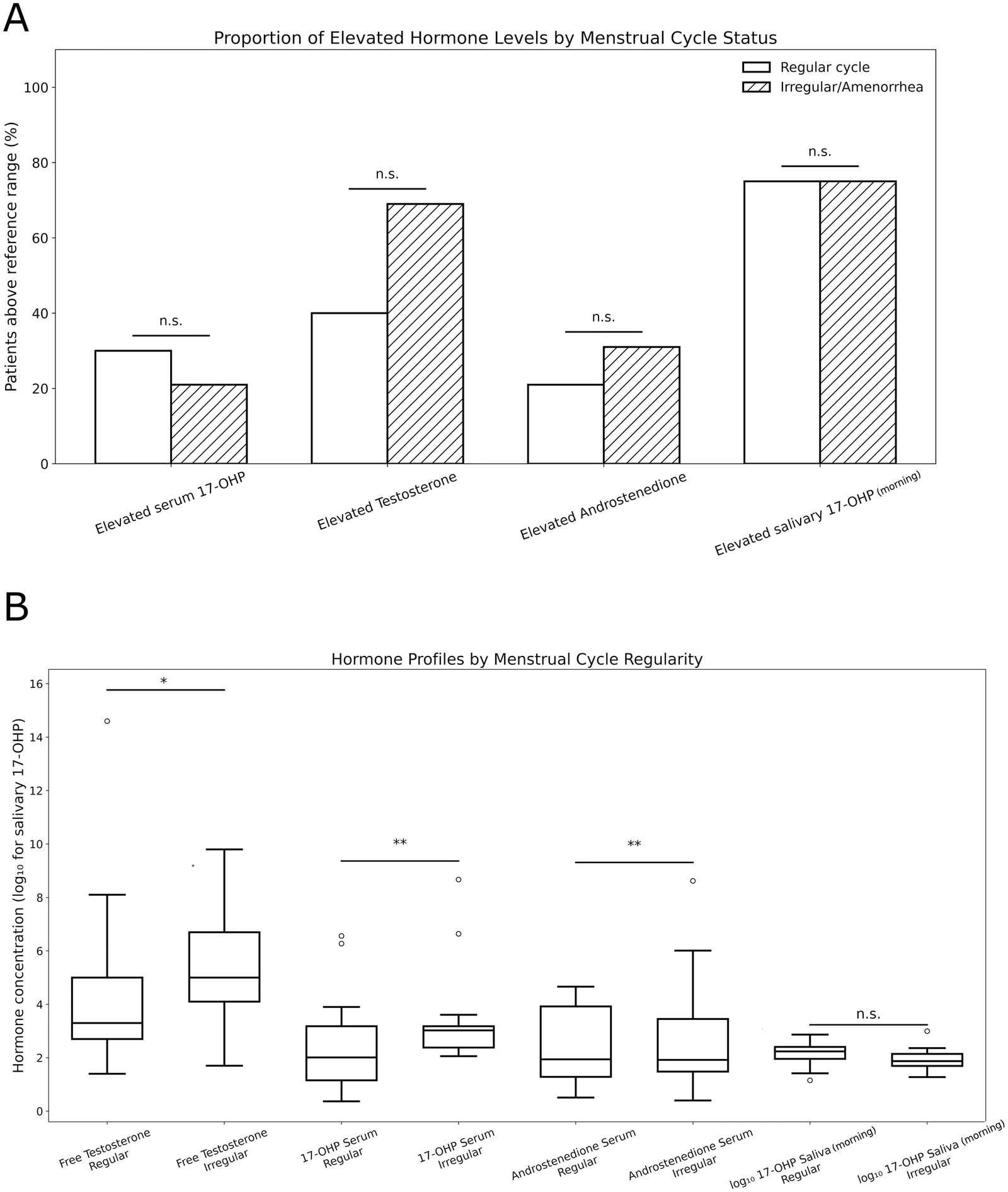
**Hormone profiles according to menstrual cycle regularity at baseline**. (**A**) Proportion of women with hormone levels above the reference range in women with regular menstrual cycles versus irregular cycles or amenorrhea (crosshatch). (**B**) Absolute hormone concentrations in women with regular and irregular cycles shown as boxplots (median, interquartile range, and range). Group comparisons were performed using the Mann–Whitney *U* test for continuous variables and Fisher’s exact test for categorical variables. 17-OHP, 17-hydroxyprogesterone. N.S., non-significant. * *P* < 0.05, **P < 0.001.

While the proportion of women with hormone levels above versus within the reference range (serum 17-OHP, early morning salivary 17-OHP, A4, and T) did not differ between women with amenorrhea or irregular cycles and those with regular menstrual cycles (Fig. 4A), absolute hormone concentrations revealed significant differences. Women with menstrual disturbances exhibited higher fT (*P* = 0.02), serum 17-OHP (*P* = 0.01), and A4 levels (*P* = 0.01) (Fig. 4B).

Among women with baseline cycle disturbance (amenorrhea or irregular cycles; n = 12 after excluding hypothalamic amenorrhea and pregnancies), ten achieved normalization to regular menstrual cycles, whereas two remained disturbed at first follow-up. Menstrual cycle improvement was not consistently associated with categorial biochemical improvement (Supplementary Fig. S3).

### Pregnancies

Of the women with a desire to conceive (n = 16), ten became pregnant during follow-up (Fig. 2). Prior to switching to or initiating MR-HC, these 10 women had been attempting to conceive unsuccessfully for a median of 28.5 months (IQR 12–46). Six of them had received prior GC therapy (HC, n = 1; prednisolone, n = 3; dexamethasone, n = 2).

In two women, the switch to MR-HC practically coincided with the onset of active attempts to conceive, whereas in the remaining four women, active attempts to conceive were initiated after the switch, with a median delay of 16 months (IQR 8.5–20.0). Across the entire cohort, accounting for the timing of active pregnancy attempts, the median time to pregnancy on MR-HC was 2.65 months (IQR 2.0–12.4).

In detail, three women conceived between baseline and V1; one of them experienced a miscarriage at 6 weeks (with a history of two previous miscarriages and prior ART use). She subsequently conceived spontaneously again 6 months later. One woman conceived between visit 2 and 3, and five conceived after visit 3 (Fig. 2). One woman who conceived within the first 3 months later had another child after 32 months while on a lower dose of MR-HC (10 mg instead of 15 mg in the evening). All pregnancies that continued during follow-up resulted in healthy live births.

Of the 10 women who became pregnant, 7 (70%) had a regular menstrual cycle at baseline, and 3 had irregular cycles (Fig. 2). In two women, normalization of the menstrual cycle was documented during follow-up (at visit 1 after 3 months), and one woman with an irregular cycle at baseline became pregnant shortly after initiation of MR-HC (Fig. 2).

Among the 10 women who became pregnant, 7 had received prior GC treatment (n = 1 hydrocortisone, n = 4 prednisolone, n = 2 dexamethasone), while 3 were treatment-naïve. Among those who conceived after prior GC treatment (n = 7), the HCeq dose remained unchanged in four women and was reduced by 5 mg in two women; in one woman the dose remained stable, and in two women it was minimally increased due to tablet availability (by 2.5 mg and 1.25 mg, respectively) (Supplementary Table S2).

### Time to pregnancy in comparison to women before the introduction of MR-HC

In comparison with a historic cohort of women with NCCAH who conceived at our center before the approval of MR-HC, from which 11 women (55%) were receiving GC treatment, the median time from active desire to conceive to pregnancy was 2.65 (2.0–12.4) versus 8 months (IQR 3.5–31.5), however due to small sample size and broad variation this difference was not statistically significant (*P* = 0.56).

The HCeq at the time of pregnancy was significantly higher in the historic cohort compared with women treated with MR-HC (median 16.9 mg (IQR 15–25) vs. 10.0 mg (IQR 10–12.5), *P* = 0.02), whereas the proportion of women requiring fertility treatment did not differ between groups (20% vs. 20%, *P* = 1.00).

### Characteristics of women who did not get pregnant

Six women did not become pregnant during follow-up (Supplementary Table S3). Of the women with a desire to conceive who did not achieve pregnancy during follow-up (median 13.5 months (IQR 6.25–23)), three had received prior GC treatment (n = 1 hydrocortisone, n = 1 prednisolone, n = 1 dexamethasone). One woman had completed only the 3-month visit so far; one underwent ovarian stimulation after V2 but did not conceive within 5 months of follow-up. One woman had completed the 12-month visit, and only the remaining two (when excluding the woman with hypothalamic amenorrhea on HRT) had an unfulfilled child wish for more than 12 months. In all but one of these women, progesterone concentrations at least at one visit indicated ovulatory cycles. The median time of an unfulfilled child-wish in this group prior to the start of MR-HC was 28.5 months (IQR 25–31).

One woman with an unfulfilled child-wish had good biochemical control on 10 mg MR-HC at 25 months of follow-up but was already 41 years old. One woman had isolated elevated 17-OHP levels and high-normal A4, one woman had isolated elevated T, and the remaining elevated 17-OHP and T levels. Dose adaptations were recommended accordingly. Among those, one woman additionally had endometriosis and a history of salpingectomy following an ectopic pregnancy, while another showed persistently elevated anti-Müllerian hormone (AMH) levels, an LH/FSH ratio >2, and a homeostatic model assessment of insulin resistance (HOMA-IR) of 4.0, suggesting a PMOS-like pathophysiological component (Supplementary Table S3).

At baseline, women who became pregnant during follow-up did not differ significantly from those who did not conceive in terms of age, BMI, AMH and baseline adrenal androgens (T, fT, A4, serum and salivary 17-OHP) (all *P* > 0.3) (Supplementary Table S4).

### Metabolic effects

Body weight showed a significant effect of time (*P* = 0.009), with a slight decrease from visit 1 to visit 3 (pairwise: *P* = 0.044) independent of GC pretreatment. Systolic blood pressure showed a significant visit × pretreatment interaction (*P* = 0.048), mainly reflecting a small decrease from visit 1 to visit 2 (pairwise: *P* = 0.033) in women with GC pretreatment. Diastolic blood pressure showed no significant changes. Fasting glucose did not change significantly. In contrast, HbA1c showed both a significant time effect (*P* < 0.001) and interaction (*P* = 0.003), with a slight increase within the normal range at visit 2 versus visit 1 (*P* = 0.019) and visit 3 (*P* = 0.042), but normalization by visit 3 (*P* < 0.001). HbA1c levels in treatment-naïve women largely converged toward the baseline values seen in pretreated women. Total triglyceride levels, total cholesterol, LDL- and HDL-cholesterol showed no significant main effects or interactions (Table 2).

**Table 2.** Changes in metabolic parameters during follow-up.

Parameter	Prior GC	Baseline	Visit 1	Visit 2	Visit 3	P time	*P* Group	*P* Time * group
Weight (kg)	No	67.10 (58.48–75.72)	66.91 (58.64–75.17)	66.10 (57.78–74.43)	69.33 (59.34–79.32)	***F*(3,10.55) = 6.58, *P* = 0.009**	*F*(1,28.32) = 0.08, *P* = 0.778	*F*(3,10.55) = 0.47, *P* = 0.709
	Yes	69.71 (63.75–75.68)	68.37 (62.68–74.06)	67.71 (61.99–73.43)	69.39 (62.63–76.14)			
SBP (mmHg)	No	128.2 (121.4–135.1)	127.6 (121.2–133.9)	125.6 (116.5–134.7)	118.2 (106.8–129.6)	*F*(3,16.7) = 2.02, *P* = 0.15	*F*(1,25) = 0.12, *P* = 0.73	***F*(3,16.7) = 3.25, *P* = 0.048**
	Yes	125.7 (120.7–130.6)	119.6 (115.1–124.0)	119.7 (113.4–126.0)	129.3 (120.8–137.9)			
DBP (mmHg)	No	83.6 (77.5–89.6)	80.9 (76.8–84.9)	80.1 (73.6–86.6)	80.1 (71.7–88.4)	*F*(3,17.1) = 0.76, *P* = 0.53	*F*(1,25.1) = 0.50, *P* = 0.49	*F*(3,17.1) = 1.13, *P* = 0.36
	Yes	78.0 (73.7–82.4)	78.2 (75.4–81.0)	77.5 (73.0–82.0)	83.0 (76.7–89.2)			
Fasting glucose (mg/dl)	No	91.89 (84.53–99.25)	90.73 (83.74–97.73)	86.57 (79.58–93.56)	83.57 (76.44–90.69)	*F*(3,11) = 0.92, *P* = 0.46	*F*(1,25.9) = 1.09, *P* = 0.31	*F*(3,11) = 1.33, *P* = 0.31
	Yes	91.84 (86.08–97.60)	90.54 (85.50–95.59)	91.95 (86.79–97.12)	92.07 (86.85–97.29)			
HbA1c (%)	No	5.17 (4.75–5.59)	5.18 (4.83–5.54)	5.41 (5.07–5.75)	5.06 (4.83–5.29)	**F(3,16.5) = 10.85, *P* < 0.001**	F(1,26.2) = 0.76, *P* = 0.39	**F(3,16.5) = 6.95, *P* = 0.003**
	Yes	5.34 (5.06–5.61)	5.42 (5.18–5.65)	5.38 (5.16–5.61)	5.34 (5.18–5.49)			
Total cholesterol (mg/dl)	No	185.5 (161.4–209.5)	182.0 (161.6–202.3)	175.1 (151.7–198.5)	186.0 (151.7–220.3)	*F*(3,5.61) = 2.82, *P* = 0.14	*F*(1,23.3) = 0.05, *P* = 0.83	*F*(3,5.61) = 0.97, *P* = 0.47
	Yes	168.5 (152.0–185.0)	182.3 (168.4–196.3)	174.2 (158.9–189.5)	194.1 (167.5–220.7)			
LDL-cholesterol (mg/dl)	No	91.1 (62.6–119.6)	100.9 (69.3–132.6)	75.4 (50.8–99.9)	88.3 (43.5–133.0)	*F*(3,12) = 1.32, *P* = 0.32	*F*(1,22.6) = 0.46, *P* = 0.50	*F*(3,12) = 0.68, *P* = 0.58
	Yes	81.9 (62.3–101.5)	80.9 (59.1–102.7)	77.4 (60.6–94.2)	64.1 (33.5–94.7)			
HDL- cholesterol (mg/dl)	No	112.9 (95.9–129.9)	107.9 (90.7–125.1)	105.4 (92.3–118.6)	116.5 (88.6–144.4)	*F*(3,8.59) = 2.54, *P* = 0.13	*F*(1,18.5) = 1.80, *P* = 0.20	*F*(3,8.59) = 1.91, *P* = 0.20
	Yes	101.8 (90.4–113.1)	105.8 (94.3–117.2)	98.1 (89.1–107.0)	110.7 (91.4–130.1)			
Triglycerides (mg/dl)	No	57.3 (39.0–75.6)	54.9 (42.4–67.5)	64.9 (40.3–89.6)	60.9 (48.8–73.0)	*F*(3,6.17) = 2.18, *P* = 0.19	*F*(1,25.6) = 0.64, *P* = 0.43	*F*(3,6.17) = 4.49, *P* = 0.054
	Yes	72.4 (59.7–85.1)	62.1 (53.6–70.5)	69.7 (52.2–87.3)	71.1 (62.4–79.7)			

Longitudinal changes were analyzed using mixed-effects models including time and prior glucocorticoid treatment as fixed effects. Patient-level repeated measures were modeled with an unstructured covariance matrix. Estimated marginal means with 95% CIs and Fisher’s least significant difference (LSD) post hoc pairwise comparisons are shown. *P* < 0.05 was considered statistically significant (bold). SBP, systolic blood pressure; DBP, diastolic blood pressure; GC, glucocorticoid; HbA1c, glycated hemoglobin.

## Discussion

This is the first study to report on the use of MR-HC in women with NCCAH. Initiating MR-HC, administered almost exclusively as a single dose at bedtime, resulted in rapid improvement in biochemical control and subsequent normalization of menstrual cycles, irrespective of prior treatment status. Among women actively attempting to conceive, most achieved pregnancy within 1 year, with a median time to conception of 2.65 months.

Overall, independent of pretreatment status, once-daily, bedtime MR-HC effectively reduced early-morning 17-OHP levels, reflecting improved overnight disease control, a cornerstone of biochemical management in CAH (Speiser *et al.*, 2018; Auer *et al.*, 2023). In classic 21OHD, adrenal steroid excess is driven primarily by the physiological nocturnal ACTH surge, which persists despite conventional glucocorticoid therapy (Debono *et al.*, 2015; Lawrence *et al.*, 2025). Although comparable circadian profiling is lacking in NCCAH, the same mechanism is thought to underlie adrenal androgen and steroid precursor excess. Consequently, suppressing the nocturnal ACTH surge rather than maintaining glucocorticoid exposure throughout the entire day appears to be the key therapeutic target. This concept is supported by the sustained reduction in testosterone levels observed in our cohort and is further reinforced by the finding that most women achieved normalization of menstrual cycles and most conceptions occurred while receiving a single bedtime dose of 10–15 mg MR-HC.

In our cohort, hormone concentrations were higher in women with menstrual disturbances compared to those without. However, adrenal androgens and androgen precursors within the reference range did not clearly correlate with menstrual cycle normalization. This suggests that biochemical normalization *per se* may not be sufficient to restore regular menstrual cyclicity.

In addition to adrenal-derived 17-OHP, A4 and testosterone disrupting the HPG-axis (Auer *et al.*, 2021, 2024; Reisch and Auchus, 2024), progesterone is known additionally to exert a direct anti-implantation effect at the endometrium. For women seeking pregnancy, follicular progesterone concentrations should therefore ideally remain <0.6 ng/ml (Reisch and Auchus, 2024). This target is consistent with physiological follicular/preovulatory progesterone concentrations in healthy women, for whom commonly reported upper reference limits range from approximately 0.5 to 1.0 ng/ml, depending on the assay used (Hoff *et al.*, 1983). However, this recommendation is based primarily on physiological considerations and has not been systematically evaluated in women with NCCAH.

Before the introduction of MR-HC into our clinical practice, we generally aimed for low-normal or even suppressed follicular progesterone and 17-OHP concentrations in women actively attempting to conceive in order to minimize periods of inadequate hormonal control throughout the day. In contrast, less stringent biochemical targets may be appropriate in women without an immediate pregnancy wish.

It should also be considered that circulating 17-OHP concentrations are highly dependent on both the timing of glucocorticoid administration and blood sampling, with fluctuations exceeding 60% within only a few hours after the morning glucocorticoid dose (Debono *et al.*, 2015). Consequently, a single serum 17-OHP measurement does not necessarily reflect overall biochemical control across the entire 24-h period. While we routinely use five-point salivary 17-OHP profiles to obtain a more comprehensive assessment of diurnal androgen precursor dynamics, the more physiological pharmacokinetic profile of MR-HC has been shown to improve the correlation between single daytime hormone measurements and overall biochemical control over 24 h (Lawrence *et al.*, 2025).

Finally, interpretation of progesterone concentrations requires consideration of menstrual cycle status. Assessment of target follicular progesterone concentrations is only meaningful in women with regular menstrual cycles and reliable cycle timing. Otherwise, mildly elevated progesterone concentrations may either indicate suboptimal adrenal suppression or simply reflect recent ovulation. Likewise, although adrenal progesterone excess is typically accompanied by elevated 17-OHP concentrations, physiological luteal-phase increases in both progesterone and 17-OHP may further complicate interpretation.

Interpretation of fertility data in NCCAH due to 21OHD is limited by the fact that most studies include symptomatic women seeking treatment, thereby introducing a bias toward a preselected group with difficulties conceiving. While overall pregnancy rates are considered normal (Feldman *et al.*, 1992; Stikkelbroeck *et al.*, 2003; Claahsen-van der Grinten *et al.*, 2006; Eyal *et al.*, 2017) and approximately half of women conceive spontaneously, the remainder require GC therapy (Birnbaum and Rose, 1984; Feldman *et al.*, 1992; Bidet *et al.*, 2010). GC therapy may particularly shorten time to conception (Bidet *et al.*, 2010) in women with >12 months of unsuccessful prior attempts (Eyal *et al.*, 2017). In our previously published multicenter cohort, median time to pregnancy was 13 months and >30% had used ovulation induction or ART (Auer *et al.*, 2024). As a subgroup analysis of this cohort treated at our center showed that median time to pregnancy was 8 months and thus shorter than in the overall cohort, we cannot exclude that comprehensive specialized care *per se*, rather than initiation of MR-HC, contributed to the rapid improvements in menstrual cyclicity and the generally short time to pregnancy observed. Because systematic data on women from that period who did not achieve pregnancy despite a desire to conceive are lacking, no conclusions can be drawn regarding overall differences in pregnancy success rates between treatment approaches.

Although not statistically significant, most probably due to the small group sizes, women treated with MR-HC who ultimately conceived did so at a median time to pregnancy of less than 3 months. Notably, this was achieved at a median MR-HC dose of only 10 mg once daily at nighttime. Excluding the woman with hypothalamic hypogonadism, only two women in our cohort experienced an unfulfilled desire to conceive for more than 12 months while receiving MR-HC.

Similarly, Bidet *et al.* (2010) reported that 83% of women with NCCAH conceived within 1 year, whereas the remaining patients required more than 12 months to achieve pregnancy. In such cases, a more detailed evaluation may be warranted to avoid overlooking non–CAH-related factors that may impair pregnancy success.

In our cohort, both women with a prolonged unsuccessful desire to conceive had regained regular menstrual cycles; however, one showed persistently suboptimal biochemical control while receiving 15 mg of MR-HC with suspected intermittent non-adherence, and the other was 41 years old, making age-related subfertility a likely contributing factor.

Another of these women with a persisting unfulfilled desire to conceive had a hormonal profile suggesting a coexistent PMOS component. While NCCAH has to be excluded before a diagnosis of PMOS can be made, an overlap between NCCAH and PMOS is well recognized (Papadakis *et al.*, 2019). 30–40% of women with NCCAH show PCOM (Dewailly *et al.*, 1986; Pall *et al.*, 2010), with even higher rates in older studies (Hague *et al.*, 1990; Levin *et al.*, 1991). In line, CYP21A2 variant carriers also have increased PMOS prevalence (Lehembre-Shiah *et al.*, 2023). Given the contribution of adrenal androgens to insulin resistance, combined NCCAH and PCOM may exacerbate menstrual dysfunction and impair fertility (Lehembre-Shiah *et al.*, 2023), and biochemical control alone may be insufficient to regulate the menstrual cycle or to normalize conception rates. Consistent with this, time to conception with GC therapy is significantly longer in NCCAH women with PCOM (Eyal *et al.*, 2017).

MR-HC was well tolerated, and only one woman discontinued therapy for personal reasons. Our preliminary data indicate a modest decrease in weight that was observed during the first 6 months, independent of pretreatment. HbA1c increased slightly in GC-naïve women, while fasting glucose remained stable. These effects were small and not sustained at 1 year, though statistical power was limited. It is well known that the interplay of GCs and hyperandrogenism contributes to altered glucose metabolism in CAH (Paizoni *et al.*, 2020). Both GC therapy and uncontrolled androgen excess can impair insulin sensitivity. While the increase in HbA1c in treatment-naïve women may reflect potentially negative effects of GCs despite the physiological doses used in all patients, one may also hypothesize that normalization of menstrual cycles may have influenced HbA1c through effects on iron metabolism and erythrocyte lifespan and therefore not necessarily reflect negative effects on glucose metabolism (Rao *et al.*, 2022).

This is the first prospective, observational study to evaluate MR-HC in a cohort of women with non-classical 21OHD under routine clinical conditions and structured follow-up.

Nevertheless, several limitations should be acknowledged. First, the absence of a contemporaneous control group receiving conventional GC therapy restricts causal inference, although contextual comparison was possible through inclusion of a historic cohort of women previously treated under comparable personal and clinical care conditions prior to the introduction of MR-HC. Second, as all participants were managed in a tertiary referral center with specialized CAH expertise, the external validity of our findings may not fully extend to less specialized clinical settings. Third, the statistical power for subgroup analyses was constrained by small sample sizes, potentially limiting detection of moderate yet clinically meaningful between-group differences. Finally, while a median follow-up of 2 years offers important longitudinal observations, this period remains too short to comprehensively assess long-term metabolic and cardiovascular outcomes.

From a clinical perspective, our findings support once-daily evening MR-HC as a promising treatment option for women with NCCAH, particularly those with menstrual disturbances or an active desire to conceive. Future prospective multicenter studies should determine whether the rapid restoration of menstrual cyclicity observed in our cohort translates into shorter time to conception, improved pregnancy outcomes, and favorable long-term metabolic safety. In addition, further studies are needed to establish evidence-based biochemical treatment targets according to patients’ reproductive goals and to optimize monitoring strategies during MR-HC therapy.

## Supplementary Material

hoag071_Supplementary_Data

## Data Availability

The datasets generated and analyzed during the current study are not publicly available but are available from the corresponding author on reasonable request.

## References

[hoag071-B1] Arlt W , Brac de la Perrière A, Hirschberg AL, Merke DP, Newell-Price JDC, Prete A, Rees DA, Reisch N, Touraine PA, Bendfeldt H et al Long-term outcomes in patients with congenital adrenal hyperplasia treated with hydrocortisone modified-release hard capsules. Eur J Endocrinol 2025;193:76–84. 40576296 10.1093/ejendo/lvaf130

[hoag071-B2] Auer MK , Minea CE, Quinkler M, Bancos I, Beuschlein F, Meyer G, Lottspeich C, Bidlingmaier M, Rieger E, Nowotny HF et al Women with congenital adrenal hyperplasia have favorable pregnancy outcomes but prolonged time to conceive. J Endocr Soc 2024;9:bvae211. 39669654 10.1210/jendso/bvae211 PMC11635451

[hoag071-B3] Auer MK , Nordenström A, Lajic S, Reisch N. Congenital adrenal hyperplasia. Lancet 2023;401:227–244. 36502822 10.1016/S0140-6736(22)01330-7

[hoag071-B4] Auer MK , Paizoni L, Neuner M, Lottspeich C, Schmidt H, Bidlingmaier M, Hawley J, Keevil B, Reisch N. 11-Oxygenated androgens and their relation to hypothalamus-pituitary-gonadal-axis disturbances in adults with congenital adrenal hyperplasia. J Steroid Biochem Mol Biol 2021;212:105921. 34058329 10.1016/j.jsbmb.2021.105921

[hoag071-B5] Bidet M , Bellanné-Chantelot C, Galand-Portier MB, Golmard JL, Tardy V, Morel Y, Clauin S, Coussieu C, Boudou P, Mowszowicz I et al Fertility in women with nonclassical congenital adrenal hyperplasia due to 21-hydroxylase deficiency. J Clin Endocrinol Metab 2010;95:1182–1190. 20080854 10.1210/jc.2009-1383

[hoag071-B6] Birnbaum MD , Rose LI. Late-onset adrenocortical hydroxylase deficiencies associated with menstrual dysfunction. Obstet Gynecol 1984;63:445–451. 6322077

[hoag071-B7] Carrière C , Nguyen LS, Courtillot C, Tejedor I, Chakhtoura Z, Bellanné-Chantelot C, Tardy V, Leban M, Touraine P, Bachelot A. Fertility and pregnancy outcomes in women with nonclassic 21-hydroxylase deficiency. Clin Endocrinol (Oxf) 2023;98:315–322. 36325983 10.1111/cen.14842

[hoag071-B8] Claahsen-van der Grinten HL , Stikkelbroeck NM, Sweep C, Hermus AR, Otten BJ. Fertility in patients with congenital adrenal hyperplasia. J Pediatr Endocrinol Metab 2006;19:677–685. 16789634 10.1515/jpem.2006.19.5.677

[hoag071-B9] Debono M , Mallappa A, Gounden V, Nella AA, Harrison RF, Crutchfield CA, Backlund PS, Soldin SJ, Ross RJ, Merke DP. Hormonal circadian rhythms in patients with congenital adrenal hyperplasia: identifying optimal monitoring times and novel disease biomarkers. Eur J Endocrinol 2015;173:727–737. 26340969 10.1530/EJE-15-0064 PMC4623929

[hoag071-B10] Dewailly D , Vantyghem-Haudiquet MC, Sainsard C, Buvat J, Cappoen JP, Ardaens K, Racadot A, Lefebvre J, Fossati P. Clinical and biological phenotypes in late-onset 21-hydroxylase deficiency. J Clin Endocrinol Metab 1986;63:418–423. 3013919 10.1210/jcem-63-2-418

[hoag071-B11] Eyal O , Ayalon-Dangur I, Segev-Becker A, Schachter-Davidov A, Israel S, Weintrob N. Pregnancy in women with nonclassic congenital adrenal hyperplasia: time to conceive and outcome. Clin Endocrinol (Oxf) 2017;87:552–556. 28731586 10.1111/cen.13429

[hoag071-B12] Feldman S , Billaud L, Thalabard JC, Raux-Demay MC, Mowszowicz I, Kuttenn F, Mauvais-Jarvis P. Fertility in women with late-onset adrenal hyperplasia due to 21-hydroxylase deficiency. J Clin Endocrinol Metab 1992;74:635–639. 1310999 10.1210/jcem.74.3.1310999

[hoag071-B13] Gordon CM , Ackerman KE, Berga SL, Kaplan JR, Mastorakos G, Misra M, Murad MH, Santoro NF, Warren MP. Functional hypothalamic amenorrhea: an Endocrine Society clinical practice guideline. J Clin Endocrinol Metab 2017;102:1413–1439. 28368518 10.1210/jc.2017-00131

[hoag071-B14] Guo X , Zhang Y, Yu Y, Zhang L, Ullah K, Ji M, Jin B, Shu J. Getting pregnant with congenital adrenal hyperplasia: assisted reproduction and pregnancy complications. A systematic review and meta-analysis. Front Endocrinol (Lausanne) 2022;13:982953. 36120452 10.3389/fendo.2022.982953 PMC9470834

[hoag071-B15] Hague WM , Adams J, Rodda C, Brook CG, De Bruyn R, Grant DB, Jacobs HS. The prevalence of polycystic ovaries in patients with congenital adrenal hyperplasia and their close relatives. Clin Endocrinol (Oxf) 1990;33:501–510. 2225492 10.1111/j.1365-2265.1990.tb03887.x

[hoag071-B16] Hirschberg AL , Gidlöf S, Falhammar H, Frisén L, Almqvist C, Nordenskjöld A, Nordenström A. Reproductive and perinatal outcomes in women with congenital adrenal hyperplasia: a population-based cohort study. J Clin Endocrinol Metab 2021;106:e957–e965. 33135723 10.1210/clinem/dgaa801

[hoag071-B17] Hoff JD , Quigley ME, Yen SSC. Hormonal dynamics at midcycle: a reevaluation. J Clin Endocrinol Metab 1983;57:792–796. 6411753 10.1210/jcem-57-4-792

[hoag071-B18] Hunter JD. Matplotlib: a 2D graphics environment. Comput Sci Eng 2007;9:90–95.

[hoag071-B19] Lawrence NR , Dawson J, Lang ZQ, Prete A, Baranowski ES, Schiffer L, Taylor AE, Brac de la Perrière A, Hirschberg AL, Juul A et al Modelling adrenal steroid profiles to inform monitoring guidance in congenital adrenal hyperplasia. EBioMedicine 2025;116:105749. 40398353 10.1016/j.ebiom.2025.105749 PMC12148605

[hoag071-B20] Lehembre-Shiah E , Gobioff S, Yeshua A, Peyser A, Maxwell S. Prevalence of congenital adrenal hyperplasia carriers in PCOS patients seeking fertility treatment. Fertil Steril 2023;120:e29.

[hoag071-B21] Levin JH , Carmina E, Lobo RA. Is the inappropriate gonadotropin secretion of patients with polycystic ovary syndrome similar to that of patients with adult-onset congenital adrenal hyperplasia? Fertil Steril 1991;56:635–640. 1915936 10.1016/s0015-0282(16)54592-0

[hoag071-B22] McKinney W. Data structures for statistical computing in Python. In: van der Walt S, Millman J (eds). *Proceedings of the 9th Python in Science Conference*; 2010, 56–61.

[hoag071-B23] Merke DP , Mallappa A, Arlt W, Brac de la Perriere A, Lindén Hirschberg A, Juul A, Newell-Price J, Perry CG, Prete A, Rees DA et al Modified-release hydrocortisone in congenital adrenal hyperplasia. J Clin Endocrinol Metab 2021;106:e2063–e2077. 33527139 10.1210/clinem/dgab051 PMC8063257

[hoag071-B24] New MI , Ghizzoni L, Meyer-Bahlburg H, Khattab A, Reichman D, Rosenwaks Z. Fertility in patients with nonclassical congenital adrenal hyperplasia. Fertil Steril 2019;111:13–20. 30611403 10.1016/j.fertnstert.2018.11.023

[hoag071-B25] Paizoni L , Auer MK, Schmidt H, Hübner A, Bidlingmaier M, Reisch N. Effect of androgen excess and glucocorticoid exposure on metabolic risk profiles in patients with congenital adrenal hyperplasia due to 21-hydroxylase deficiency. J Steroid Biochem Mol Biol 2020;197:105540. 31730799 10.1016/j.jsbmb.2019.105540

[hoag071-B26] Pall M , Azziz R, Beires J, Pignatelli D. The phenotype of hirsute women: a comparison of polycystic ovary syndrome and 21-hydroxylase-deficient nonclassic adrenal hyperplasia. Fertil Steril 2010;94:684–689. 19726039 10.1016/j.fertnstert.2009.06.025

[hoag071-B27] Papadakis G , Kandaraki EA, Tseniklidi E, Papalou O, Diamanti-Kandarakis E. Polycystic ovary syndrome and NC-CAH: distinct characteristics and common findings. A systematic review. Front Endocrinol (Lausanne) 2019;10:388. 31275245 10.3389/fendo.2019.00388 PMC6593353

[hoag071-B28] Rao LV , Pratt GW, Bi C, Kroll MH. Large-scale retrospective analyses of the effect of iron deficiency anemia on hemoglobin A1c concentrations. Clin Chim Acta 2022;529:21–24. 35167841 10.1016/j.cca.2022.02.005

[hoag071-B29] Reisch N , Auchus RJ. Pregnancy in congenital adrenal hyperplasia. Endocrinol Metab Clin North Am 2024;53:391–407. 39084815 10.1016/j.ecl.2024.05.005

[hoag071-B30] Speiser PW , Arlt W, Auchus RJ, Baskin LS, Conway GS, Merke DP, Meyer-Bahlburg HF, Miller WL, Murad MH, Oberfield SE et al Congenital adrenal hyperplasia due to steroid 21-hydroxylase deficiency: an Endocrine Society clinical practice guideline. J Clin Endocrinol Metab 2018;103:4043–4088. 30272171 10.1210/jc.2018-01865 PMC6456929

[hoag071-B31] Stikkelbroeck NML , Hermus ARM, Braat DD, Otten BJ. Fertility in women with congenital adrenal hyperplasia due to 21-hydroxylase deficiency. Obstet Gynecol Surv 2003;58:275–284. 12665708 10.1097/01.OGX.0000062966.93819.5B

[hoag071-B32] Waskom ML. Seaborn: statistical data visualization. Joss 2021;6:3021.

