# Supplementary material for: Once-daily, late-night, modified-release hydrocortisone rapidly normalizes menstrual cyclicity in women with non-classic congenital adrenal hyperplasia
Source: Hum Reprod Open. 2026 Aug 5;2026(3):hoag071. doi: 10.1093/hropen/hoag071 (PMC13549656; doi:10.1093/hropen/hoag071)
Supplement: Supplementary file 1 [file hoag071_supplementary_data.docx]

**Supplementary Figure S1. Frequency of elevated hormone levels at baseline according to prior glucocorticoid therapy**

The figure shows the proportion of women with serum 17-hydroxyprogesterone (17-OHP), androstenedione and testosterone concentrations above the female reference range at baseline, stratified by prior glucocorticoid (GC) therapy. P-values were calculated using Fisher's exact test. * p < 0.05; p < 0.01; p < 0.001

**Supplementary Figure S2. Menstrual cycle changes stratified by prior glucocorticoid pretreatment status**

Among the five women with prior GC treatment who presented with irregular cycles (n = 5) or amenorrhea (n = 1), five regained a regular cycle within the first three months and one regained a regular cycle after at least six months (visit 1 data missing). Among the patients without prior GC treatment and irregular menstrual cycles (n = 6), one woman got pregnant within 3 months, two regained a regular cycle within three months, two within six months and one by at last twelve month (information on menstrual cycle was missing in between). Among the three treatment-naïve women with secondary amenorrhea, one regained a regular menstrual cycle within the first three months, one still had an irregular cycle at six months and later opted for an oral contraceptive pill, and the woman with secondary amenorrhea due to hypothalamic hypogonadism following anorexia nervosa remained amenorrheic and was started on hormone replacement therapy after 12 months. mo: months. OCP: Oral contraceptive therapy. Irreg: irregular menstrual cycle.

**Supplementary Figure S3. Hormonal status according to improvement in menstrual cyclicity.**

Changes in serum 17-hydroxyprogesterone (17-OHP), morning salivary 17-OHP, androstenedione, and testosterone are shown according to improvement or persistence of menstrual cycle disturbances. Hormone concentrations were categorized as normal-normal, high-normal, high-high, or normal-high according to baseline and follow-up measurements.

**Supplementary Table S1. Biallelic CYP21A2 genotype combinations in the study cohort.**

| **Genotype Combination** | **n** |
| --- | --- |
| p.Val282Leu / p.Val282Leu | 6 |
| p.Ile173Asn / p.Val282Leu | 3 |
| p.Pro453Ser / Deletion | 3 |
| c.293-13C>G (I2G) / p.Val282Leu | 3 |
| p.Pro30Leu / p.Val282Leu | 3 |
| c.293-13C>G (I2G) / p.Pro30Leu | 2 |
| p.Val282Leu / Deletion | 2 |
| p.Val282Leu / p.Gln318* | 1 |
| p.Val282Leu / p.Gly454* | 1 |
| p.His63Leu / p.Arg133Cys | 1 |
| Complex allele† / p.Val282Leu | 1 |
| Frameshift (c.331delG) / p.Val282Leu | 1 |
| p.Ile172Asn / p.Val281Leu | 1 |

**† Complex allele:** promoter variants (c.–126C>A; c.–110T>C; c.–103A>G)

**Supplementary Table S2. Characteristics of women who became pregnant during follow-up**

| **Age (years)** | **Genotype** | **Unfulfilled child wish before switching (months)** | **Time to pregnancy (months)** | **Follow-up (months)** | **Time to conception after switch** | **Menstrual cycle at baseline** | **Menstrual cycle before pregnancy** | **Pretreatment** | **MR-HC baseline** | **MR-HC before pregnancy** | **Biochemical status at baseline** | **Biochemical status at last visit before pregnancy** |
| --- | --- | --- | --- | --- | --- | --- | --- | --- | --- | --- | --- | --- |
| 42 | V282L / Del | 12 | 1.5 | 15 | Directly pregnant (1st pregnancy) | regular | - | None | 0-0-10 mg | 0-0-10 mg | **Morning 17-OHP saliva ↑** | Directly pregnant |
|  |  |  |  |  |  |  |  |  |  |  | **A4 ↑** |  |
|  |  |  |  |  |  |  |  |  |  |  | **17-OHP (Serum)↑** |  |
|  |  |  |  |  |  |  |  |  |  |  | **T ↑** |  |
|  |  |  |  |  |  |  |  |  |  |  | AMH↔ |  |
| 28 | V281L / V281L | 1 | 1.6 | 8 | Directly pregnant | regular | - | Dexamethasone 0-0-0.0125mg | 0-0-10 mg | 0-0-10 mg | Morning 17-OHP saliva ↔ | Directly pregnant |
|  |  |  |  |  |  |  |  |  |  |  | A4 ↔ |  |
|  |  |  |  |  |  |  |  |  |  |  | 17-OHP (Serum) ↔ |  |
|  |  |  |  |  |  |  |  |  |  |  | T ↔ |  |
|  |  |  |  |  |  |  |  |  |  |  | AMH  **(missing)** |  |
| 27 | P453S / Del | - | 2.3 | 40 | — | irregular | regular | None | 0-0-10 mg | 0-0-10 mg | Morning 17-OHP saliva ↔ | Morning 17-OHP saliva↔ |
|  |  |  |  |  |  |  |  |  |  |  | A4 ↔ | A4 ↔ |
|  |  |  |  |  |  |  |  |  |  |  | 17-OHP (Serum) ↔ | 17-OHP (Serum)↔ |
|  |  |  |  |  |  |  |  |  |  |  | **T ↑** | T ↔ |
|  |  |  |  |  |  |  |  |  |  |  | AMH↔ |  |
| 30 | I173N / V282L | 0 | 12.4 | 21 | — | irregular | regular | None | 0-0-10 mg | 0-0-10 mg | Morning 17-OHP saliva ↔ | Morning 17-OHP saliva **(missing)** |
|  |  |  |  |  |  |  |  |  |  |  | A4 ↔ | A4 ↔ |
|  |  |  |  |  |  |  |  |  |  |  | 17-OHP (Serum) **(missing)** | 17-OHP (Serum) ↔ |
|  |  |  |  |  |  |  |  |  |  |  | **T ↑** | **T ↑** |
|  |  |  |  |  |  |  |  |  |  |  | AMH↔ |  |
| 27 | V282L / G454* | - | 15 | 38 | — | regular | regular | Prednisolone 2-0-1mg | 0-0-10 mg | 0-0-10 mg | Morning 17-OHP saliva ↔ | Morning 17-OHP saliva ↔ |
|  |  |  |  |  |  |  |  |  |  |  | A4 ↔ | A4 ↔ |
|  |  |  |  |  |  |  |  |  |  |  | 17-OHP (Serum) ↔ | 17-OHP (Serum) ↔ |
|  |  |  |  |  |  |  |  |  |  |  | T ↔ | T ↔ |
|  |  |  |  |  |  |  |  |  |  |  | AMH **(missing)** |  |
| 33 | H63L / R133C | 46 | 12.4 | 43 | — | regular | regular | Prednisolone 4-0-1mg | 0-0-15 mg | 0-0-15 mg | Morning 17-OHP saliva  **(missing)** | **Morning 17-OHP saliva↑** |
|  |  |  |  |  |  |  |  |  |  |  | A4 ↔ | A4 ↔ |
|  |  |  |  |  |  |  |  |  |  |  | 17-OHP (Serum) ↔ | 17-OHP (Serum) ↔ |
|  |  |  |  |  |  |  |  |  |  |  | T ↔ | T ↔ |
|  |  |  |  |  |  |  |  |  |  |  | AMH↔ |  |
| 34 | V282L / Del | - | 14 | 43 | — | regular | regular | HC 10-5-0mg | 0-0-15 mg | 5-0-10 mg | Morning 17-OHP **(missing)** | Morning 17-OHP saliva ↔ |
|  |  |  |  |  |  |  |  |  |  |  | A4 ↔ | A4 ↔ |
|  |  |  |  |  |  |  |  |  |  |  | 17-OHP (Serum) ↔ | 17-OHP (Serum) ↔ |
|  |  |  |  |  |  |  |  |  |  |  | T ↔ | T ↔ |
|  |  |  |  |  |  |  |  |  |  |  | AMH  **(missing)** |  |
| 33 | V282L / V282L | - | 22.1 | 26 | — | regular | regular | Prednisolone 2-0-0mg | 0-0-10 mg | 0-0-10 mg | **Morning 17-OHP saliva ↑** | **Morning 17-OHP saliva↑** |
|  |  |  |  |  |  |  |  |  |  |  | A4 ↔ | A4 ↔ |
|  |  |  |  |  |  |  |  |  |  |  | 17-OHP (Serum) ↔ | **17-OHP (Serum) ↑** |
|  |  |  |  |  |  |  |  |  |  |  | T ↔ | T ↔ |
|  |  |  |  |  |  |  |  |  |  |  | AMH↔ |  |
| 36 | V282L / I2G | 94 | 14 | 27 | — | regular | regular | Dexamethasone 0-0-0.0125mg | 0-0-10 mg | 0-0-15 mg | **Morning 17-OHP saliva ↑** | Morning 17-OHP saliva ↔ |
|  |  |  |  |  |  |  |  |  |  |  | **A4 ↑** | **A4 ↑** |
|  |  |  |  |  |  |  |  |  |  |  | **17-OHP (Serum) ↑** | **17-OHP (Serum) ↑** |
|  |  |  |  |  |  |  |  |  |  |  | **T ↑** | **T ↑** |
|  |  |  |  |  |  |  |  |  |  |  | AMH↔ |  |
|  |  |  |  |  |  |  |  |  |  |  |  |  |
| 27 | G111Vfs*21 / V282L | 12 | 2.1 | 39 | — | irregular | irregular | Prednisolone 2-0-1mg | 0-0-15 mg | 0-0-15 mg | Morning 17-OHP (saliva)  **(missing)** | Morning 17-OHP (saliva)  **(missing)** |
|  |  |  |  |  |  |  |  |  |  |  | **A4 ↑** | **A4 ↑** |
|  |  |  |  |  |  |  |  |  |  |  | 17-OHP (serum) ↔ | 17-OHP (serum) ↔ |
|  |  |  |  |  |  |  |  |  |  |  | **T ↑** | **T ↑** |
|  |  |  |  |  |  |  |  |  |  |  | AMH↔ |  |

Abbreviations: MR-HC: modified-release hydrocortisone; HC: hydrocortisone; T: testosterone; A4: androstenedione; 17-OHP: 17-hydroxyprogesterone; AMH: anti-Müllerian hormone; Del: CYP21A2 deletion; ↔: within the reference range; ↑: above the reference range.

**Supplementary Table S3. Characteristics of women who did not become pregnant during follow up**

| **Age (years)** | **Genotype** | **Unfulfilled child wish before switching (months)** | **Follow-up (months)** | **Menstrual cycle at baseline** | **Comorbidities** | **Menstrual cycle last** | **Pretreatment** | **MR-HC baseline** | **MR-HC last** | **Biochemical status at baseline** | **Biochemical status at last visit** |
| --- | --- | --- | --- | --- | --- | --- | --- | --- | --- | --- | --- |
| 31 | I173N / V282L | 12 | 17 | Amenorrhea |  | regular | Dexamethasone 0-0-0.25 | 0-0-10mg | 0-0-15mg | Morning 17-OHP (saliva) ↔ | Morning 17-OHP (saliva) ↔ |
|  |  |  |  |  |  |  |  |  |  | A4 ↔ | A4 ↔ |
|  |  |  |  |  |  |  |  |  |  | **17-OHP (serum)↑** | **17-OHP (serum)↑** |
|  |  |  |  |  |  |  |  |  |  | T ↔ | T ↔ |
|  |  |  |  |  |  |  |  |  |  | AMH ↔ | AMH↔ |
| 41 | P30L / V282L | 29 | 25 | regular |  | regular | Prednisolone 1.5-0-0 | 0-0-10mg | 0-0-10mg | Morning 17-OHP (saliva) ↔ | Morning 17-OHP (saliva)  **(missing)** |
|  |  |  |  |  |  |  |  |  |  | A4 ↔ | A4 ↔ |
|  |  |  |  |  |  |  |  |  |  | 17-OHP (serum)↔ | 17-OHP (serum)↔ |
|  |  |  |  |  |  |  |  |  |  | T ↔ | T ↔ |
|  |  |  |  |  |  |  |  |  |  | AMH↔ | AMH↔ |
| 37* | P453S / Del | 31 | 5 | irregular | Endometriosis and history of salpingectomy due to ectopic pregnancy | regular | None | 0-0-10mg | 0-0-10mg | Morning 17-OHP (saliva) ↔ | Morning 17-OHP (saliva) ↔ |
|  |  |  |  |  |  |  |  |  |  | A4↔ | A4↔ |
|  |  |  |  |  |  |  |  |  |  | 17-OHP (serum) *↔* | 17-OHP (serum)↔ |
|  |  |  |  |  |  |  |  |  |  | ***T ↑*** | **T ↑** |
|  |  |  |  |  |  |  |  |  |  | AMH↔ | AMH↔ |
| 31 | V282L / I2G | 25 | 3 | regular |  | regular | None | 0-0-10mg | 0-0-10mg | **Morning 17-OHP (saliva*)* ↑** | **Morning 17-OHP (saliva) ↑** |
|  |  |  |  |  |  |  |  |  |  | **A4↑** | A4↔ |
|  |  |  |  |  |  |  |  |  |  | **17-OHP (serum) ↑** | **17-OHP (serum) ↑** |
|  |  |  |  |  |  |  |  |  |  | **T ↑** | **T ↑** |
|  |  |  |  |  |  |  |  |  |  | **AMH↑** | AMH↔ |
| 30 | V282L / V282L | 57 | 37 | Amenorrhea | Hypothalamic amenorrhea | Hyopthalamic hypogonadism, put on HRT after 12 months | HC 15-0-0 | 0-0-15mg | 0-0-15mg | Morning 17-OHP (saliva) ↔ | Morning 17-OHP (saliva) ↔ |
|  |  |  |  |  |  |  |  |  |  | A4↔ | A4↔ |
|  |  |  |  |  |  |  |  |  |  | 17-OHP (serum) ↔ | 17-OHP (serum) ↔ |
|  |  |  |  |  |  |  |  |  |  | T ↔ | T ↔ |
|  |  |  |  |  |  |  |  |  |  | **AMH↑** | AMH↔ |
| 29* | P30L / V282L | 28 | 10 | irregular |  | regular | None | 0-0-10mg | 0-0-20mg | **Morning 17-OHP (saliva) ↑** | Morning 17-OHP (saliva) ↔ |
|  |  |  |  |  |  |  |  |  |  | **A4↑** | A4↔ |
|  |  |  |  |  |  |  |  |  |  | **17-OHP *(serum)* ↑** | **17-OHP (serum*)* ↑** |
|  |  |  |  |  |  |  |  |  |  | **T ↑** | **T↑** |
|  |  |  |  |  |  |  |  |  |  | **AMH↑** | **AMH↑** |

Abbreviations: MR-HC: modified-release hydrocortisone; HC: hydrocortisone; T: testosterone; A4: androstenedione; 17-OHP: 17-hydroxyprogesterone; AMH: anti-Müllerian hormone; Del: CYP21A2 deletion; ↔: within the reference range; ↑: above the reference range.

**Supplementary Table S4. Baseline characteristics according to conception status during follow-up**

| **Variable** | **Pregnant (N=10)** | **Not Pregnant (N=6)** | **p-value** |
| --- | --- | --- | --- |
|  | **Median (IQR)** | **Median (IQR)** |  |
| **Age (years)** | 25.0 (21.0–32.5) | 31.0 (29.0–37.0) | 0.883 |
| **Weight (kg, M0)** | 72.3 (55.9–81.9) | 66.0 (61.7–80.7) | 0.482 |
| **BMI (kg/m²)** | 25.9 (19.7–30.9) | 23.8 (23.4–31.9) | 0.565 |
| **AMH (ng/mL)** | 3.55 (1.75–8.85) | 5.71 (1.77–9.36) | 0.830 |
| **Testosterone (ng/dL)** | 77.5 (37.3–95.8) | 63.0 (45.0–124.0) | 0.380 |
| **Free testosterone (pg/mL)** | 4.65 (2.20–7.45) | 5.85 (2.45–11.0) | 0.344 |
| **17-OHP serum (ng/mL)** | 2.98 (2.04–6.21) | 2.46 (2.06–3.90) | 0.314 |
| **Androstenedione (ng/mL)** | 1.72 (1.33–4.09) | 1.60 (1.40–4.58) | 0.558 |
| **Morning 17-OHP saliva (ng/dL)** | 134 (75–230) | 49.2 (24.4–114.0) | 0.337 |

Values are median (interquartile range). P-values were calculated using the Mann–Whitney U test. AMH: anti-Müllerian hormone; 17-OHP: 17-hydroxyprogesterone; IQR: interquartile range.
